# A Technology-Enhanced Intervention for Violence and Substance Use Prevention Among Young Black Men: Protocol for Adaptation and Pilot Testing

**DOI:** 10.2196/43842

**Published:** 2023-05-01

**Authors:** Chuka Emezue, Niranjan S Karnik, Blaine Reeder, Michael Schoeny, Rickey Layfield, Amie Zarling, Wrenetha Julion

**Affiliations:** 1 Department of Women, Children and Family Nursing Rush University College of Nursing Chicago, IL United States; 2 Department of Psychiatry Institute for Juvenile Research University of Illinois Chicago Chicago, IL United States; 3 Sinclair School of Nursing University of Missouri-Columbia Columbia, MO United States; 4 Department of Community, Systems and Mental Health Nursing Rush University College of Nursing Chicago, IL United States; 5 Urban Male Network Vice President of Programming Chicago, IL United States; 6 Department of Human Development and Family Studies Iowa State University Ames, IA United States

**Keywords:** youth, violence, adolescent health, mindfulness, digital health, usability, Black, African American, men, mobile app

## Abstract

**Background:**

Black boys and men from disinvested communities are disproportionately survivors and perpetrators of youth violence. Those presenting to emergency departments with firearm-related injuries also report recent substance use. However, young Black men face several critical individual and systemic barriers to accessing trauma-focused prevention programs. These barriers contribute to *service avoidance*, the exacerbation of violence recidivism, substance use relapse, and a revolving-door approach to prevention. In addition, young Black men are known to be digital natives. Therefore, technology-enhanced interventions offer a pragmatic and promising opportunity to mitigate these barriers, provide vital life skills for self-led behavior change, and boost service engagement with vital community resources.

**Objective:**

The study aims to systematically adapt and pilot-test Boosting Violence-Related Outcomes Using Technology for Empowerment, Risk Reduction, and Life Skills Preparation in Youth Based on Acceptance and Commitment Therapy (BrotherlyACT), a culturally congruent, trauma-focused digital psychoeducational and service-engagement tool tailored to young Black men aged 15-24 years. BrotherlyACT will incorporate microlearning modules, interactive safety planning tools for risk assessment, goal-setting, mindfulness practice, and a service-engagement *conversational agent* or chatbot to connect young Black men to relevant services.

**Methods:**

The development of BrotherlyACT will occur in 3 phases. In phase 1, we will qualitatively investigate barriers and facilitators influencing young Black men’s willingness to use violence and substance use prevention services with 15-30 young Black men (aged 15-24 years) who report perpetrating violence and substance use in the past year and 10 service providers (aged >18 years; any gender; including health care providers, street outreach workers, social workers, violence interrupters, community advocates, and school staff). Both groups will be recruited from community and pediatric emergency settings. In phase 2, a steering group of topic experts (n=3-5) and a youth and community advisory board comprising young Black men (n=8-12) and service providers (n=5-10) will be involved in participatory design, alpha testing, and beta testing sessions to develop, refine, and adapt BrotherlyACT based on an existing skills-based program (Achieving Change Through Values-Based Behavior). We will use user-centered design principles and the Assessment, Decision, Administration, Production, Topical, Experts, Integration, Training, and Testing framework to guide this adaptation process (phase 2). In phase 3, a total of 60 young Black men will pilot-test the adapted BrotherlyACT over 10 weeks in a single-group, pretest-posttest design to determine its feasibility and implementation outcomes.

**Results:**

Phase 1 data collection began in September 2021. Phases 2 and 3 are scheduled to start in June 2023 and end in September 2024.

**Conclusions:**

The development and testing of BrotherlyACT is a crucial first step in expanding an evidence-based psychoeducational and service-mediating intervention for young Black men involved in violence. This colocation of services shifts the current prevention strategy from telling them *why* to change to teaching them *how*.

**International Registered Report Identifier (IRRID):**

PRR1-10.2196/43842

## Introduction

### Background

Youth violence and substance use co-occur as a public health double threat. Youth violence encompasses a range of behaviors, including dating violence, actual or threatened violence, physical fighting, web-based and school bullying, threats and assault with weapons, homicide, and firearm-related and gang-related violence [1,2]. Similarly, substance use is linked to violent incidents, even as persistent substance use (of marijuana and alcohol) is associated with youth violence perpetration and victimization [3,4]. Although the evidence is conflicting [4,5], youth violence and substance use share transdiagnostic risk and protective factors [4,6] and underlying mechanisms that cross-cutting preventative measures can address. Moreover, these socially costly issues are linked to multiple adverse physical, social, and health effects, such as low quality of life, subpar academic and social functioning, mental health disorders, and suicide risk [4].

Young Black men and boys from disinvested communities are disproportionately survivors, perpetrators, and witnesses of youth violence and homicide [7-9]. Recent Centers for Disease Control and Prevention data reports homicide as the primary cause of death for Black boys and men (aged 10-24 years), and firearms were involved in 94% of these incidents [9-11]. During the pandemic, firearm homicide rates in the United States rose to its highest level since its peak in 1994 [12]. Black boys and men aged 10 to 44 years saw the largest increase in these adjusted rates from 2019 to 2020, followed by Native American individuals, aged 25 to 44 years [7,12]. Consequently, Black youth are 14 times more likely and Hispanic youth are 3 times more likely than White youth to die from firearm-related violence [12].

More than 700,000 urban youths (aged 10-24 years) present to emergency departments (EDs) each year with nonfatal assault-related injuries [4,13,14]. Approximately 20% of these youths report substance use within 3 hours of an assault-related injury [3,14,15]. Despite this, service gaps continue to hamper violence and substance use prevention efforts for underserved, urban, and low-income youth. Outside the juvenile and criminal justice systems, racial and ethnic minority youth report critical barriers to obtaining behavioral health services across the continuum of prevention, treatment, and recovery. These barriers include but are not limited to neighborhood disadvantage, racism, homelessness or temporary housing, unemployment, discrimination, peer and gang pressures, transportation issues, program fees, the lack of culturally relevant services, and competing demands on time to attend clinic or group-based rehabilitation sessions [16-18].

Similarly, violence prevention programs in hospital EDs, schools, juvenile justice facilities, child welfare systems, faith-based and street outreach initiatives continue to have substantial wraparound effects in reducing violence [19,20]. However, in resource-limited communities, these programs lack 24/7 staff coverage, follow-up services, and trauma-informed training and report high employee turnover [16-18,21]. Furthermore, injured young Black men perceive hospital violence intervention programs—and other formal institutions—as places of interface with law enforcement and thus avoid using these services [16,22-24], even though EDs are increasingly recognized as a critical intervention venue for youth at risk for future violent injury [4].

Similar to contexts where these services exist, sociostructural barriers can also impede youth willingness to use them. These barriers include and are exacerbated by experiences of police bias and violence, treatment stigma [16,17,25-27], racial trauma, and a historic mistrust of legal and medical systems linked to the systemic discrimination and overt criminalization of Black men and boys [16,28-30]. In addition, the language (and culture) of formal therapy centering on Western-centric curricula [31] and gender norms that frame help-seeking as a *weakness* have also been identified by young Black men as impediments to service use despite the high prevalence rates of trauma exposure among these underserved groups [32,33].

These barriers have created substantial service gaps [17]. In addition, they have exacerbated treatment inequities, leaving young Black men involved in violence in underserved communities to adopt ineffective coping techniques, such as service avoidance or *opting out* [22]. Service avoidance takes the form of deliberate attempts to avoid or disengage from formal treatment or rehabilitation programs perceived to be associated with *feds* or that place them *in the system* [22], whereas *opting out* from preventative programs can manifest as early dropouts, noncompliance, nonparticipation, tardiness, and absenteeism even when the court system mandates program completion. Consequently, persistent service-avoidant behaviors contribute to violence recidivism, substance use relapse, and a revolving-door approach to prevention. Therefore, improving youth access to and trust in existing violence and substance use prevention interventions is critical.

### Technology-Enhanced Interventions to Reduce Barriers to Co-occurring Violence and Substance Use Prevention

Technology-enhanced interventions can empower underserved and low-income youth to overcome some of these barriers [18]. Studies show that young Black men favor digital interventions as digital natives in a *tech-saturated* world [34-37]. Even after controlling for age, parent education, and family composition (single vs 2-parent households), Black youth have higher cell phone ownership rates and internet use (ie, hours per day) than Hispanic and White adolescents [36]. Therefore, technology-enhanced interventions can simultaneously mitigate these barriers and broaden the access to trauma-focused therapies. From a prevention science standpoint, engaging at-risk young Black men in this digital landscape—by meeting them where they are—is a promising and pragmatic approach to early prevention of substance-related violence [37-39]. This strategy can potentially reduce service avoidance while boosting the availability, accessibility, and personalization of evidence-based interventions [37-39]. Notably, technology-enhanced interventions can serve the “treatment curious” (those at early risk who have not sought out formal services but who can benefit from a low-stakes digital tool that highlights the risks and consequences of youth violence) and the “treatment veterans” (or returning citizens who are chronically involved *in the system* and familiar with violence and substance use services). Innovative public health and equity-focused interventions promoting health equity and positive service engagement for at-risk and underserved young Black men are needed to address the gaps and barriers identified previously.

This study aimed to systematically adapt and pilot-test Boosting Violence-Related Outcomes Using Technology for Empowerment, Risk Reduction, and Life Skills Preparation in Youth Based on Acceptance and Commitment Therapy (BrotherlyACT), a culturally congruent, trauma-focused digital intervention tailored to Black boys and men aged 15 to 24 years. BrotherlyACT will incorporate microlearning modules, interactive safety planning tools for risk assessment, goal-setting, mindfulness practice, and a service-engagement *conversational agent* or chatbot to connect young Black men to relevant services.

### Overview of BrotherlyACT

**Figure 1:**
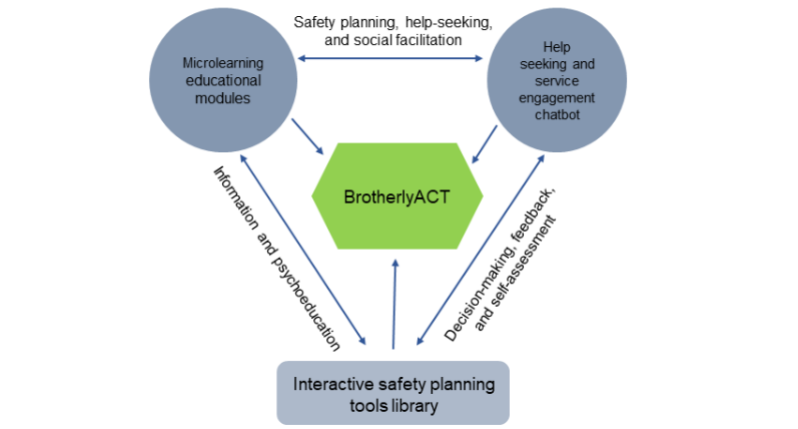
Boosting Violence-Related Outcomes Using Technology for Empowerment, Risk Reduction, and Life Skills Preparation in Youth Based on Acceptance and Commitment Therapy (BrotherlyACT) components.

To address these gaps and barriers, we developed a protocol for adapting and pilot-testing BrotherlyACT. It is envisioned as a multilevel, multicomponent, web-delivered, and app-delivered intervention to promote psychoeducation, safety planning, and service-engagement self-efficacy to reduce the risk and effects of co-occurring youth violence and substance use among low-income Black boys and men (aged 15-24 years). BrotherlyACT will comprise three main components (as depicted in Figure 1): (1) microlearning psychoeducational modules (or brief lessons framed as learnable *life skills* in an interactive curriculum) organized around the 6 fundamental acceptance and commitment therapy (ACT) principles (intrapersonal focus); (2) a safety planning tools library with resources to facilitate risk assessment, goal-setting and tracking, and mindfulness practice (interpersonal focus); and (3) an SMS text messaging–styled service-engagement chatbot or *conversational agent* to promote help-seeking, self-advocacy, and access to a curated list of national and local violence and substance use prevention programs and services (interactional focus). The need for these three components emerged out of interest from young Black men and service providers working with teens involved in violence to help them (1) develop self-efficacy to practice help-seeking while accessing trusted services on their own, (2) create a supportive motivational climate for underserved youth to feel comfortable seeking help, and (3) identify safe people and resources in their communities.

### Theoretical Rationale

BrotherlyACT is based on several theoretical and conceptual models. The intervention’s psychoeducational modules will adapt an existing theory-driven and skills-based manualized intervention (Achieving Change Through Values-Based Behavior [ACTV]) with motivational, informational, and behavioral activation strategies [40-45]. These brief or microlearning psychoeducational modules will cover topics on help-seeking, substance use, conflict resolution, nonretaliation, healthy relationships, gun and gang refusal skills, mindfulness (putting a *brake* on emotional reactivity), and positive future orientation and coping.

The original ACTV curriculum comprises 5 modules designed for violence reduction with incarcerated men and women using group sessions [38-44]. In addition to feedback from young Black men and service providers, the other components of BrotherlyACT were informed by the Capability, Opportunity, and Motivation to Change Behavior model [46], which proposes 3 elements that are vital to sustainable behavior change: capability, opportunity, and motivation [46]. *Capability* refers to an individual’s abilities and skills, such as physical, cognitive, or technical skills, and considers situational and background risk factors that precipitate youth violence. *Opportunity* refers to the presence or availability of resources and opportunities that enable the individual to engage in a particular behavior, such as access to information, social support, or resources, and practice self-direction and decision-making. *Motivation* refers to the individual’s desire or willingness to engage in the behavior, which may be influenced by factors such as attitudes, beliefs, values, or goals. Capability and opportunity jointly influence motivation [46], contributing to lasting behavior change. Taken together, the Capability, Opportunity, and Motivation to Change Behavior model helped to clarify individual, community, and structural risk factors (eg, service avoidance and emotional avoidance) that may be targeted by BrotherlyACT.

We hypothesize that through improved behavioral and cognitive skills and service-engagement self-efficacies, BrotherlyACT can reduce key psychological avoidant mechanisms (emotional avoidance and service avoidance) and increase motivation, capability (eg, self-efficacy to create and use a safety plan), and opportunity (eg, knowledge about service options). These improved conditions will, in turn, reduce the risk and effects of violence and co-occurring substance use among low-income young Black men involved in violence.

### ACTV Approach

ACTV is an evidence-based psychotherapy based on ACT [47-52]. ACT is a third-generation cognitive behavioral therapy (CBT) based on the philosophy of functional contextualism and Relational Frame Theory [53]. ACT differs from other CBT-based approaches because it is based on a unified model of human behavior change and psychological growth. It is focused on helping individuals develop a different relationship with their thoughts and emotions instead of trying to change them directly. For example, CBT aims to help individuals identify and change negative or irrational thoughts, whereas ACT holds that pain and discomfort are a fact of life—something we must become comfortable with if we wish to live a happy, fulfilled life. ACT aims to increase *psychological flexibility* or “the process of contacting the present-moment fully as a conscious human being and persisting or changing behavior in the service of chosen values” and reduce *emotional avoidance* (unwillingness or perceived inability to remain in contact with difficult internal experiences and efforts to delay gratification and live a value-based lifestyle) [47-52]. Therefore, ACT encourages flexibly responding with openness and acceptance of our thoughts and emotions rather than trying to directly change their form or frequency and then shifting our attention (with practice) to what is most important in our lives. Of note, >20 meta-analyses and >900 randomized trials, with 12,477 participants, have shown that ACT is effective for a wide range of target conditions, with effect sizes ranging from small to medium for depression, anxiety, substance abuse, and chronic pain and small to large effects for increasing psychological flexibility in general [53,54].

ACTV was developed by a coauthor (AZ) and has been rigorously tested in multiple randomized effectiveness trials, feasibility studies, and pilot studies in collaboration with the Iowa Department of Corrections [38-44]. A series of effectiveness trials compared ACTV with the traditional Duluth or CBT model. A 1-year postintervention comparison of community-based ACTV vs the Duluth and CBT models (widely used globally in rehabilitation programs for abusive men) found that ACTV significantly reduced new charges, domestic assault charges, and violent charges for men who were mandated to complete a domestic violence intervention program (N=3474) at 12 months after treatment [42]. In addition, this effect was maintained for both program completers and dropouts [40]. A recent 5-year survival analysis of ACTV participants corroborated a previous study with 1-year outcomes, showing ACTV participants being less likely to acquire new violent or domestic abuse charges at year 5 (*χ*^2^_1_=6.8, *P*=.009) but with no difference in general criminal charges between ACTV and Duluth or CBT participants (*χ*^2^_1_=0.3, *P*=.60) [40]. In addition, control participants acquired new charges faster than intervention participants [40]. Although effective for reducing partner and criminal violence, ACT (and ACTV) has not been adapted for cross-cutting violence and substance use prevention with at-risk youth or for digitally enhanced delivery.

However, trauma-focused therapies, such as ACTV, remain inaccessible to youth who are economically disadvantaged and belong to a racial and ethnic minority group (eg, Black and Latinx youth) [54]. These interventions are typically developed, adapted, and evaluated with White, high-income populations [55-57]. In addition, White youth who are involved in violence are more likely than their non-White counterparts to be diverted to rehabilitative therapy [58]. In comparison, young Black men who are involved in violence are substantially more likely to be transferred to more punitive alternatives (eg, transferred to adult court) or other forms of detention [58]. Interventions addressing these issues similarly use Western-centric theories for individualistic violence prevention and emphasize racial and socioeconomic distinctions between treatment facilitators, program curricula, and men referred to these programs, resulting in a *therapeutic mismatch* [31].

### User-Centered Design for Technology-Enhanced Interventions

User-centered design (UCD) is a general term for design methodologies in which end users are involved in the iterative process to shape the design of an intervention and contexts for its implementation [59-61]. UCD strategies can be put into practice quite fast, enabling us to swiftly determine user needs and create appropriate interventions to address these needs.

UCD methods are important, as they can uncover new information that is not revealed by conventional techniques, improve user experience, and ensure that we obtain representative participant response to match a predetermined usability requirement. In addition, users develop a sense of ownership for the product [59]. Contextual interviews, think-aloud protocols, cognitive walk-throughs, semistructured cognitive interviews, and acceptability or feasibility surveys are some of the UCD techniques we will use with key stakeholders as we develop BrotherlyACT.

The development of BrotherlyACT using UCD principles reimplements the design and adaptation methodologies from our previous studies with young men who are involved in violence and affected by trauma [34,62]. In more recent studies, we have used UCD methods in the design of a mobile clinical decision support app for urinary tract infection in long-term care [63,64] and in understanding the usability and function of consumer-grade wearable devices [65].

Several iterations of BrotherlyACT will be produced and tested for usability. Early studies indicate a need for cross-cutting interventions for violence co-occurring with other risky behaviors. Young men in these studies confirmed the suitability of, and the potential to explore further, the use of digital methodologies as a primary prevention tool [34]. Participants listed the following as intervention *must-haves*: individualized feedback, compelling firearm injury testimonials, inspiring tales of other young men who have successfully avoided violence and substance use, humanlike chat capabilities, actual stories and violence-related testimonials, pictures and videos of people similar to them, and mechanisms to monitor behavior change. This feedback substantially informed the proposed BrotherlyACT intervention [34].

## Methods

### Overview

The development of BrotherlyACT will occur in 3 phases. In phase 1, we will collaborate with Black boys and men (aged 15-24 years) and service providers (aged >18 years; any gender) to identify social and structural barriers and motivators influencing youth violence and substance use prevention. Black boys and men (aged 15-24 years; n=25-30) and their service providers (n=10) will be recruited from clinical (pediatric ED) and community-based settings in the West and South sides of Chicago—2 areas with historically high violence burden and persistent economic disadvantages, but with much promise and a wealth of community-based programs [66-69]. Sessions will include participatory focus groups, in-depth one-to-one interviews, and wish listing activities (ie, listing intervention must-haves).

**Figure 2:**
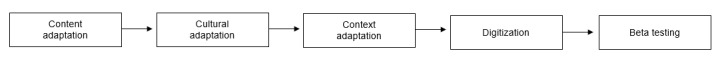
Outline of study phases.

In phase 2, we will use content and cultural adaptations of the ACTV curriculum using the 8-stage Assessment, Decision, Administration, Production, Topical, Experts, Integration, Training, and Testing (ADAPT-ITT) framework [70,71]. Finally, in phase 3, we will assess the feasibility and acceptability of BrotherlyACT. Overall, 60 young Black men (not part of phase 1 or 2) will remotely pilot-test BrotherlyACT for 10 weeks to determine feasibility and implementation outcomes. An outline of the study stages is shown in Figure 2.

### Ethics Approval

All study phases have been approved by the Rush University Medical Center institutional review board (IRB; Office of Research Affairs number 21122902-IRB01). In addition, we have received recruitment-only IRB approval from the Stroger Cook County Hospital (study 22-087R). Each participant will provide informed consent before recruitment into the study and receive a copy of the signed form as a PDF. They will also be informed that their participation is voluntary and that they may withdraw from the study at any time. Furthermore, permission will be sought to audio-record and take notes during the interviews. All study data will be deidentified and kept confidential according to IRB protocols at designated study sites, with Rush IRB being the primary study site.

### Study Design, Participants, and Recruitment

#### Phase 1—Risk and Needs Assessment

##### Overview

**Figure 3:**
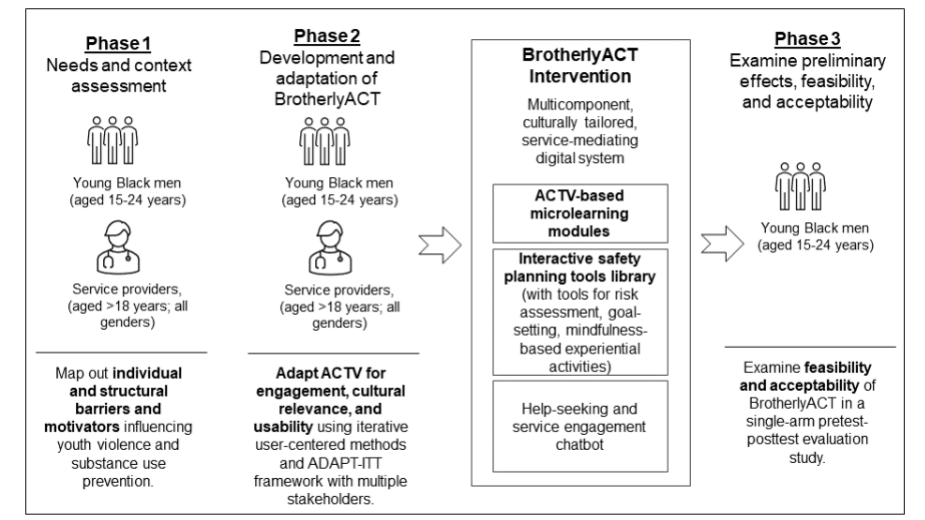
Outline of study design. ACTV: Achieving Change Through Values-Based Behavior; ADAPT-ITT: Assessment, Decision, Administration, Production, Topical, Experts, Integration, Training, and Testing; BrotherlyACT: Boosting Violence-Related Outcomes Using Technology for Empowerment, Risk Reduction, and Life Skills Preparation in Youth Based on Acceptance and Commitment Therapy.

This phase will involve formative, qualitative research using individual web-based interviews and focus group discussions with key informants (young Black men and service providers). This needs elicitation phase will identify social and structural barriers and motivators influencing youth violence and substance use prevention services among Black boys and men (aged 15-24 years) and their service providers (including but not limited to health care providers, street outreach workers, social workers, violence interrupters, community advocates, and school staff). In separate focus groups and individual interviews, we will include community barrier mapping and graphic facilitation sessions (ie, displaying ideas expressed during meetings on a whiteboard with text and imagery). Young Black men and service providers will describe (1) sociostructural targets to improve young Black men engagement with violence prevention services, (2) culturally salient needs, (3) knowledge of existing resources (eg, where, how, and why young Black men seek assistance), and (4) preference for digital behavior change interventions. In addition, we will age-stratify all interviews (15-17 years and 18-24 years) to account for differences in risk taking, peer culture, developmental age, and self-identity. The service providers and young Black men will not interact in the same sessions. Instead, separate sessions will be scheduled for service providers so that young Black men may openly communicate their experiences and account for developmental, background, and age differences without bias. Sessions will be conducted in person, via phone, or via secure video chat (Zoom; Zoom Video Communications Inc). The outline of the study design is shown in Figure 3.

##### Study Setting

Study sessions will occur concurrently, with Zoom, phone (phases 1 and 2), and in-person options for participation (phase 3). Only in Chicago, where the study researchers are based, will participants be able to undergo in-person procedures in pediatric EDs and community-based centers, if they prefer. We have chosen to start with young Black men in 1 major high-risk city (Chicago) to establish proof of concept before expanding to other cities with a high violence burden (Detroit, Baltimore, Camden, and Oakland) in the future.

##### Consent and Assent Process

Black boys aged 15 to 17 years will assent, and Black men aged 18 to 24 years will consent. Participants can electronically assent or consent on a Health Insurance Portability and Accountability Act–compliant e-consent platform. The informed e-consent form will include information about the study’s aim, procedures, confidentiality, and risks and benefits of participation and the safety and risk mitigation protocols. Alternatives for contacting the study team (ie, text, calls, and emails) will accommodate participant needs and account for digital divides, internet bandwidth issues, and digital health equity concerns (eg, privacy and data safety concerns). We will not collect written documentation of youth assent and consent to safeguard the participants’ confidentiality. We will waive parental consent as this study poses low risk, according to federal laws (45 Code of Federal Regulations 46), Section 116 (d) [72]. Waiving parental consent does not affect the rights and welfare of our participants. Low rates of young Black men enrollment when parental consent is mandated have impeded previous studies.

Moreover, young Black men with low risk (less substance use; little to no violent history) are oversampled when parental consent is mandated (individuals with high risk or great use of violence and substances are less likely to join owing to this reason). Waiving parental consent may reduce sampling biases and dishonest reporting by participants, as adolescent participants may fear their parents discovering their participation in the study. Ineligible participants will be redirected to the end of the survey, where they will find resources (eg, crisis helplines and suicide hotlines) to help young Black men obtain the needed services. In addition, once the e-consent form is submitted, participants can print or save a copy of the informed assent and consent form before moving on to the web-based survey. Importantly, there is no more than minimal risk involved in completing the brief web-based sociodemographic questionnaire, and the participants’ responses will remain completely anonymous (no IP addresses will be automatically logged in REDCap [Research Electronic Data Capture; Vanderbilt University]).

##### Consent Process for Service Providers

Service providers will be required to verbally consent at the beginning of each session. Service providers will be informed about the voluntary nature of this study, that all questions are optional, and that the session will be audio-recorded. As a part of the consenting process, service providers will be informed that they will not have access to any young Black men’s data, even if they come from the same organization.

##### Data Analysis for Phase 1

Focus groups and interviews will be audio-recorded and transcribed verbatim by an independent transcriptionist. Transcripts from the study sessions will be recorded (with permission after informed consent), transcribed verbatim, and analyzed by 2 research team members using qualitative description, low-abstraction, and low-inference approach to closely interpret the data [73-75]. Qualitative description is ideal for describing phenomena of the participants’ experience and language and is well suited for needs and risk assessment with susceptible populations [73-75]. After sorting, rearranging, and comparing codes, we will form categories and subcategories by pooling codes based on commonality, divergence, and logical parsimony [60,61,76]. Primary themes will be identified based on data generalizations and supplemented by participant excerpts [73,74]. Qualitative analysis software will be used to develop codes and a codebook. Approximately 10% of all transcripts will be double coded as pilot transcripts and reviewed for intercoder reliability or consistency to ensure high coder agreement (κ>0.80). In addition, *wish list* data will be analyzed according to a multilevel content analysis using an inductive six-phase process [76]: (1) coding of data sources (ie, focus group and interview transcripts), (2) reflexive note taking and bracketing, (3) cross-examination to discern similarities and variations in data, (4) observing commonalities and differences among the data, (5) identifying stable generalizations from the data, and (6) comparison of results with established literature.

##### Inclusion and Exclusion Criteria

Black boys and men invited to participate are eligible if they identify as male, irrespective of sexual orientation; identify as Black or African American; are aged between 15 and 24 years (inclusive); report having internet access; and are able to assent or consent to participation. We have received an IRB waiver of parental consent, as this study presents minimal risk. Those who are actively suicidal or homicidal, not medically stable, intoxicated during recruitment, and cognitively unable to provide informed consent; have impairments (such as emergent injuries); or are in police custody or incarcerated during the study enrollment will be excluded. Service providers will be adults (1) aged >18 years (all genders), (2) with experience in working with racial and ethnically diverse youths, (3) of any race or ethnicity, and (4) who are able to speak and read in English.

##### Recruitment Plan

Recruitment will be based on the Strategies to Recruit, Engage, and Retain Children in Behavioral Health Risk Factor Studies (REACH) model [77]. Strategies will include distributing flyers in high-traffic areas frequented by adolescents (eg, youth-serving agencies, festivals, churches, not-for-profit organizations, sports centers, reentry resource fairs, and barbershops). Our partner organizations will assist in recruitment using channels they deem appropriate for reaching the youth they serve. Partner organizations include The Urban Male Network and the Chicago Black Therapists Network (a network of 140 Black therapists). Both programs support underserved Black youth with mentoring and mental health services. We will also recruit through clinical research platforms, such as ResearchMatch (funded by the National Institutes of Health) [78] and The New Normal. We will work with 2 urban pediatric EDs: John H Stroger, Jr Hospital of Cook County (IRB approved), a level-1 trauma hospital that treats approximately 120,000 adults and children annually; and the 62-bed pediatric ED at Rush University Medical Center. Social media will support broadcast recruitment (via Instagram and Facebook advertisements). Service providers will be recruited via direct email to relevant organizations, word of mouth, and snowballing. Study materials will refer to the *Youth Health and Safety Study*.

A community champion familiar with the West and South sides of Chicago will advise and facilitate recruitment in these communities. Similarly, clinical research assistants in each ED or in-patient trauma unit will enroll participants presenting with assault-related or violence-related injuries who meet our eligibility criteria. All study staff will be trained in research compliance, motivational interviewing, trauma-informed research and data collection, and sensitivity training. After obtaining consent, young Black men will complete a survey on REDCap to assess their eligibility. Participants’ compensation will be graded, ranging from US $25 to US $40, with the amount increasing for each study session completed. Service providers will receive US $50 for each session they complete.

##### Retention Plan

Similarly, we will use the REACH model [77] to maximize participant retention. These strategies will include the following: (1) obtaining the names, phone numbers, and email addresses or SMS text messages of several (at least 3) individuals (ie, friends, parents, and siblings) as alternate safe contacts to keep in touch with participants; (2) after each study communication, the participant will be provided with study contact information; (3) gift card compensation and train or bus passes will be provided for in-person meetings; and (4) a secured database for organizing follow-up contacts. When the app is developed and ready for testing (ie, phase 3), retention plans will include offering participants access to a password-protected study website with instructions on how to safely use the app and maintaining periodic contact throughout the study to assess for safety and confirm planned or unplanned changes to contact information.

#### Phase 2—Adapt and Optimize BrotherlyACT

##### Overview

Pertinent themes from phase 1 will inform the adaptation of the ACTV curriculum into a microlearning format in BrotherlyACT. This stage will be focused on iteratively developing all 3 components of BrotherlyACT tailored to young Black men affected by violence and substance use. The microlearning modules will draw on the evidence-based ACTV intervention by adapting, modifying, or adding modules directly relevant to young Black men’s unique experiences in noninstitutionalized contexts. UCD principles and the 8-stage ADAPT-ITT framework will guide this adaptation process. The ADAPT-ITT framework is an 8-stage sequential guide to intervention adaptation, particularly in resource-poor settings [70,71]. Overall, 2 phases of stakeholder adaptations will occur at this stage. Stage 1 will focus on content and cultural adaptations and alpha testing. Then, stage 2 will comprise context adaptations and beta testing. UCD efforts will be informed and guided by our previous study using this methodology.

##### Stage 1

#### Overview

This phase of adaptation and optimization will involve *content* and *cultural* adaptations (ie, ADAPT-ITT stages 1-5; refer to Table S1 in Multimedia Appendix 1) and will conclude with alpha testing with topic experts and a multidisciplinary steering group (n=8-10; including the codeveloper of ACTV). This steering group will alpha test using semistructured cognitive interviews and several acceptability and feasibility questionnaires, including the 10-item System Usability Scale (SUS) [79]. The SUS can heuristically capture the perceived ease of use of BrotherlyACT (usability) and learnability (the ease with which users understand the intervention curriculum and layout). The SUS score is stable, with as few as 5 participants testing it. The SUS score must average at least 68 out of 100 (68 is the universal average acceptable score) [79,80]. The SUS is highly reliable (Cronbach α=.90), with scores >70 indicating *good* usability [80].

#### Content Adaptation

In sessions lasting 60 to 90 minutes, adaptation will focus on altering the noncore components of the ACTV curriculum, including language; simplifying ACTV modules while retaining its therapeutic values; and providing examples, case studies, and metaphors relevant to young Black men. Adaptation will also refine BrotherlyACT to determine which core components must be maintained or excluded (eg, therapeutic jargon). Specifically, we will systematically adapt ACTV skills-based modules into a paper-based microlearning curriculum (draft 0) that will be evaluated and refined independently.

Participants will be taught and will read each of the modules in the ACTV curriculum. Following each module, debriefing sessions will be completed to assess acceptability. Participants will then discuss and recommend (1) vital components to be retained, excluded, emphasized, or condensed to ensure that the adapted curriculum is appropriate for young Black men based on age, context, and community setting; (2) how to structure the module’s learning objective in line with Bloom Taxonomy (to improve knowledge, life skills self-efficacy, and motivation to learn); (3) the modules’ voice, tone, and pacing; (4) engaging formats for lesson practice and rehearsal (eg, true or false factoids, card sorting exercises, multichoice questions, and quizzes); and (5) module length. The paper-based curriculum will be drafted based on this feedback using Microsoft Word.

Next, adaptation plans will focus on how BrotherlyACT’s clinical goals (eg, life skills self-efficacy) will map to BrotherlyACT’s other in-app components and pragmatic features (safety planning toolkit and service-engagement chatbot). Overall, curriculum adaptations will consider logistical issues, such as intervention workflow, and what point-of-care organizational and community capacities are required for BrotherlyACT to be successfully implemented.

#### Cultural Adaptation

This step is vital, as identity, racial socialization, and culture are central themes in minority youth decision-making to seek and use prevention services and to facilitate great sustained engagement in treatment. Culturally adapted interventions must consider racial socialization messaging, beliefs, cultural values, contexts, and racial barriers that affect young Black men. We will also plan to incorporate culturally salient factors (ie, spirituality, Black masculinity, family support, fatherhood, a community, or *village mentality*) into draft 0. These factors influence help-seeking among young Black men [81]. In addition, we will incorporate relevant media (eg, videos of reformed young Black men), providing cultural and community icons as role models, and positive ethnic pride messaging (eg, messages countering the myth that Black boys are prone to violence). Cultural adaptation will necessitate modifying elements of draft 0 based on 8 cultural domains (language, contexts, symbols, metaphors, content, concepts, goals, methods, and context) through the lens of the Ecological Validity Model [82].

##### Stage 2

This phase of adaptation and optimization will include *context* adaptations, digitization, and *beta testing* of BrotherlyACT by a youth and community advisory board (YCAB).

#### Context Adaptation

The paper-based curriculum (*draft 0*) will be optimized into a digitized curriculum (ie, interactive web-accessible and app-accessible format) as part of BrotherlyACT (*draft 1*). Using UCD principles that incorporate youth needs [59-61,72,83], the YCAB (young Black men [n=8-12] and service providers [n=5-10]) will alpha test draft 1 for contextual acceptability, esthetics, understandability, relevance, and cultural salience. This wireframe version will not have color or graphics and will concentrate on content flow, functionality, and priority using sketches and layouts of interfaces with clickable components, buttons, and dropdowns. This stage of adaptation will increase BrotherlyACT’s suitability to be digitized.

Next, our technology partner (Hekademeia Research Solutions [84,85]) will build a prototype of BrotherlyACT (*draft 2*), beginning with the adapted curriculum. This version will be functional and reflective of the final intervention. The original ACTV is a standardized manual in a predetermined sequence of modules across 24 weeks, which may restrict its flexibility in digital format. Therefore, BrotherlyACT will allow for the nonsequential deployment of interactive micromodules (that can be completed in 5-7 minutes) in response to the urgency of youth violence and the immediate skills needed and to fulfill real-world needs in high-violence, high-vigilance contexts. We will reconvene confirmatory focus groups with our YCAB to inform a finalized version of BrotherlyACT (*draft 3—*a high-fidelity prototype).

#### Beta Testing

The YCAB will beta test draft 3 in 2 consecutive sessions (broken into 2 rounds to reduce the cognitive load on participants) and build on findings from iterative sessions. Each session will last 30 to 90 minutes to identify usability problems early, quickly, and economically to find areas for improvements and to recommend content and features to retain or remove (ie, with sufficient features and functionality for testing). The YCAB will click portions (eg, buttons on the interface) on study iPads or personal phones. They will observe, rate, and clarify content tone and esthetic issues using a semiscripted usability protocol, including *cognitive walk-throughs* (to identify content and phrases not understood or interpreted as intended in modules and app pages) based on scripted cognitive tasks. *Theater testing* will be used to clarify their cognitive processes as they react to questions about content (“what comes to mind when the app asks you if you want to redo a module?”), paraphrase or summarize app-related elements (eg, text), and identify technical issues (eg, glitching or slow-loading pages) [71].

#### Phase 3—Clinical Trial

##### Overview

Overall, 60 young Black men (not part of phases 1 and 2) will be recruited to a single-group, pretest-posttest study of the finalized version of BrotherlyACT. This pilot test will determine its early feasibility outcomes (the feasibility of recruitment and retention methods; feasibility of module co-design with youth by observing fidelity to and deviations from the study protocol; completion of outcome measures; and satisfaction, appropriateness, cultural relevance, feasibility, and acceptability of the intervention). We will also ascertain the implementation outcomes for a large trial (ie, adherence, sustainability, and usability).

Assessments will be taken at baseline (T0) and after the intervention (T1) at 10 weeks, with SMS text message prompts to complete modules and study sessions. Given the exploratory nature of this study and its focus on feasibility, acceptability, and implementation outcomes, phase 3 data analyses will not be designed to have a specified level of statistical power. Nonetheless, with sample size of N=60, pretest-posttest correlation of 0.60, and α^2^ (tailed)=.05, we estimate 80% power to detect a pretest-posttest change of Cohen *d*≥0.33 (a small to medium effect). We will use mean imputation on the missing items in the event of participant loss. Participants who leave the study will be compared with those who stay to identify any differences in baseline and follow-up data for descriptive purposes and to determine if data are missing at random.

##### Data Analysis for Phase 3

Pretest and posttest scores will be compared to see if participants improved, stayed the same, or deteriorated on each outcome scale. Data will be checked for missing data patterns, distributions of outliers, and variable distributions to ensure that they meet the assumptions of planned analyses. For baseline characteristics, descriptive statistics will be generated. We are limited in attributing postintervention change without a control group to the intervention. Thus, the effects observed are preliminary. All statistical analyses will be conducted using SAS. Initial data analysis will be performed to check for data quality, including allowable ranges and errors.

Generalized linear models will analyze the pretest and posttest survey measures using repeated measures analysis. As this pilot study is exploratory, we will use a *P* value <.05 to denote significant findings but not adjust for multiple tests, given the pilot nature of the study. Exploratory analyses will examine differences in rates of pretest-posttest change by baseline characteristics, including recruitment site (pediatric ED vs community). To test such moderation effects, terms for the moderator and for the moderator × time (before and after) interaction will be added to the models described previously. Significant interactions will be graphed to aid interpretation. At the end of the 10-week study period, participants will complete exit interviews via the web individually and will complete the SUS. Additional open-ended process interview questions will focus on young Black men’s perceptions of BrotherlyACT and the research experience. As part of this study, responses to exit interview questions will be recorded with permission and thematically analyzed in a qualitative phase before being combined with pretest and posttest findings.

## Results

Phase 1 data collection began in September 2021. Phases 2 and 3 are scheduled to start in June 2023 and end in September 2024. The entire study is expected to be completed by the end of 2025.

## Discussion

### Expected Findings

Using a mixed methods study format, subjective and objective feasibility and acceptability data will be compared. This will ensure that survey data can be quantified and contribute to our qualitative understanding of the curriculum’s practicality and acceptability.

Few antiviolence technology-enhanced interventions exist, and even fewer studies have studied the acceptability, usability, and engagement of a life skills and mindfulness coaching intervention for co-occurring youth violence and substance use among young Black men. The extent to which existing technology-enhanced interventions are used for violence prevention has been limited to supporting the work of street outreach workers [86]. We expect that BrotherlyACT will prove to be acceptable and usable by young Black men affected by violence. Furthermore, findings from the development of BrotherlyACT will be useful for a future definitive trial. This trial will validate an expanded version of BrotherlyACT across clinical and community-based settings for young Black men in Chicago and other cities with a high prevalence of youth violence. Clinical outcomes will be determined after we evaluate how well BrotherlyACT works (ie, reduction in violence and substance use, perceived likelihood of involvement in violence and problematic behaviors, self-efficacies to plan for a positive future, use of ACT and life skills, and likelihood of using service options). The success of this intervention in different deployment contexts (eg, in a hospital vs a community setting or web-based vs face-to-face setting) will be evaluated as part of the implementation analysis. BrotherlyACT will be tested (1) with referred (vs recruited) young Black men, (2) with an organic deployment by service providers (vs research staff), and (3) in comparison with conventional care in future effectiveness trials.

On the basis of previous application of qualitative methods [34] and UCD [34,59,62], we anticipate that our study will highlight the benefits of technology-enhanced modalities for addressing motivation and self-efficacy to reduce and prevent co-occurring violence and substance use among at-risk young Black men. Studies so far support an association between substance use and violence [4,5], with a consensus that substance use can increase both the frequency and severity of violence [4-6]. A common substance implicated in violent experiences when misused is alcohol, with the suggestion that eliminating hazardous drinking would reduce general violence by 44% [5]. Such findings have led many researchers to focus on developing integrated intimate partner violence and substance use interventions [4-6].

However, this study is the first stage in developing and evaluating a technology-enhanced intervention for reducing co-occurring violence and substance use among Black boys and men aged 15 to 24 years in a high-violence setting. We are using an existing theory-driven and evidence-based intervention (ie, ACTV) that has been proven to reduce violence. This approach eliminates the need for expensive additional investments in developing a new intervention from other data sources, which may face significant feasibility issues and require considerable time. Extending ACTV to noninstitutionalized juvenile samples exposed to youth violence is a logical next step. Addressing our research objectives squarely will drive innovation in violence prevention and identify settings, conditions, and limitations for situations where digital solutions are inapplicable or even detrimental.

Furthermore, crime prediction technologies, digital surveillance tactics, and facial recognition software have all been known to target and penalize Blackness [86]. Therefore, we approach this study through a transformative justice lens, mindful of how technology affects Black communities. We hold ourselves and our team accountable in our quest for ethical and dignified technology-enhanced interventions. We remain responsible to young Black men, who will support this process and, eventually, use BrotherlyACT.

### Strengths and Limitations

This study and our approach have some limitations. As our study only includes young Black men, we cannot generalize our results to other groups of young men (such as Hispanic; tribal; and lesbian, gay, bisexual, transgender, queer, and more youth) who are disproportionately affected by violence and substance use. Furthermore, using a single sample group for pretest and posttest will not infer causative relationships and will limit the causal attribution related to the proposed intervention. However, our goal at this stage is to develop and pilot-test BrotherlyACT for preliminary feasibility and acceptability. We have evaluated the power of our sample size using suggestions and guidelines for conducting pilot studies contained in the existing literature.

Nevertheless, it is possible that our sample size is very small, which could influence the results we receive. Regarding cultural adaptation of the intervention to young Black men, it can be challenging to present diverse groups with a unified view of *culture*. Some participants may find that traditional values conflict with their convictions or that generic cultural values may not adequately capture the particulars of their own cultural experience. Therefore, we intend to develop BrotherlyACT’s architecture to be adaptable to user requirements and capable of rapid, iterative adjustments. Moreover, young Black men who have perpetrated violence and use illicit substances are a self-selecting population that may not feel comfortable reporting their substance use and histories of violence, thus necessitating the use of novel recruitment tactics.

### Conclusions

At the individual level, studies show that life skills are a vital tool and coping strategy for adolescents in high-trauma contexts. Life skills have been used for decades to prevent violence and aggression in adolescents, particularly those in their early to middle adolescence. However, there is compelling evidence that low-income urban youth have few opportunities to observe, learn, or practice the actionable skills required to deal with the heightened levels and severity of the stressors they face. Accordingly, there is a need for combining multilevel strategies, such as psychoeducation and safety planning (at the individual level), with service engagement at the community level. There is also a dire need for easily accessible and youth-endorsed interventions that allow young people to acquire and practice these abilities as they plan for a values-based life. Improving Black adolescents’ access to skill-building resources and connecting them to already existing and trusted services is a critical and urgent public health need, the absence of which has resulted in a revolving-door approach to youth violence and substance use prevention.

## Appendix

Multimedia Appendix 1 Assessment, Decision, Administration, Production, Topical, Experts, Integration, Training, and Testing framework.

## Abbreviations

ACT: acceptance and commitment therapy
ACTV: Achieving Change Through Values-Based Behavior
ADAPT-ITT: Assessment, Decision, Administration, Production, Topical, Experts, Integration, Training, and Testing
BrotherlyACT: Boosting Violence-Related Outcomes Using Technology for Empowerment, Risk Reduction, and Life Skills Preparation in Youth Based on Acceptance and Commitment Therapy
CBT: cognitive behavioral therapy
ED: emergency department
IRB: institutional review board
REACH: Strategies to Recruit, Engage, and Retain Children in Behavioral Health Risk Factor Studies
REDCap: Research Electronic Data Capture
SUS: System Usability Scale
UCD: user-centered design
YCAB: youth and community advisory board

## Data Availability

Study data sets will be deposited in publicly available repositories (where available and appropriate), as dictated by our funders, or presented in the main paper or additional supporting files whenever possible. Using a community-engaged approach ensures that the research findings are owned by the community, sustainable, and widely disseminated. Therefore, the dissemination of results in the community will be considered along with community input on how Boosting Violence-Related Outcomes Using Technology for Empowerment, Risk Reduction, and Life Skills Preparation in Youth Based on Acceptance and Commitment Therapy can be integrated into existing violence prevention programs, policies, and resources for schools, clinics, and community-based settings serving young Black men. Furthermore, service providers can use the adapted Boosting Violence-Related Outcomes Using Technology for Empowerment, Risk Reduction, and Life Skills Preparation in Youth Based on Acceptance and Commitment Therapy curriculum (paper or digital version) as a brief intervention resource to help at-risk and underserved adolescents in various contexts.
