# Supplementary material for: A Technology-Enhanced Intervention for Violence and Substance Use Prevention Among Young Black Men: Protocol for Adaptation and Pilot Testing
Source: JMIR Res Protoc. 2023 May 1;12:e43842. doi: 10.2196/43842 (PMC10186193; doi:10.2196/43842)
Supplement: Multimedia Appendix 1 [file resprot_v12i1e43842_app1.docx]

Multimedia Appendix 1. Assessment, Decision, Administration, Production, Topical, Experts, Integration, Training, and Testing framework.

| **Adaptation and pilot testing of BrotherlyACT using ADAPT-ITT** | | |
| --- | --- | --- |
| 1 | Assessment | - Consider the culturally relevant and context-specific needs of YBM's risk profile for violence and substance use using in-depth interviews and focus groups; analyze the qualitative data collected |
| 2 | Decision | - In collaboration with stakeholders, decide on aspects of ACTV to BrotherlyACT for YBM, - Plan how to structure and present the adapted intervention. |
| 3 | Adaptation | - Use novel adaptation frameworks to aid in the adaptation process |
| 4 | Production | - Produce paper-based and digital curriculum |
| 5 | Topical Experts | - Share curriculum and adaptation results with multi-stakeholder pilot testing, YBM, service providers, topic experts to provide feedback |
| 6 | Integration | - Improve and refine adaptation, incorporate feedback and review changes to produce finalized curriculum |
| **Planned post-implementation phase** | | |
| 7 | Training | - Train service providers and target users who will use and recommend BrotherlyACT (i.e., dissemination). |
| 8 | Testing | - Collect pre- and post- effectiveness data to determine feasibility and acceptability, - Examine the effectiveness of the adapted BrotherlyACT in a full-scale randomized control trial |
